# Delayed Subaponeurotic Fluid Collection in an Infant Following Instrumental Delivery: A Case Report

**DOI:** 10.7759/cureus.103214

**Published:** 2026-02-08

**Authors:** Adnan Khan, Zarbakhta Ashfaq, Ahmad Reshad Payenda, Maimoona Zubair, Wadood Khan

**Affiliations:** 1 Neurosurgery, Northwest General Hospital and Research Centre, Peshawar, PAK; 2 Medicine, Northwest General Hospital and Research Centre, Peshawar, PAK; 3 Anaesthesia, Northwest General Hospital and Research Centre, Peshawar, PAK; 4 General Surgery, Northwest General Hospital and Research Centre, Peshawar, PAK

**Keywords:** conservative management, delayed subaponeurotic fluid collection, infant scalp swelling, instrumental delivery, neonatal scalp lesions, pediatric case report, subgaleal hemorrhage differential

## Abstract

We report the case of a 70-day-old male infant who presented with a progressively enlarging, soft, fluctuant swelling over the vertex of the scalp, first noticed on day 43 of life. The infant was born via instrumental vaginal delivery following prolonged labor but had no signs of trauma or scalp swelling at birth. The child remained asymptomatic, with no fever, irritability, or feeding difficulties, and demonstrated normal neurological development. Physical examination revealed a non-tender, fluctuant swelling without overlying skin changes or signs of infection. A thorough differential diagnosis included caput succedaneum, cephalohematoma, subgaleal hemorrhage, and delayed subaponeurotic fluid collection (DSFC). Given the absence of systemic symptoms and neurological deficits, a provisional diagnosis of DSFC was made, and the infant was managed conservatively. The swelling was monitored with scheduled follow-ups, and spontaneous resolution was anticipated. This case emphasizes the importance of recognizing DSFC in the differential diagnosis of scalp swellings in infants and highlights the effectiveness of conservative management in the absence of complications.

## Introduction

Subaponeurotic (subgaleal) fluid collection in neonates and infants represents a rare but important clinical entity. This occurs in the potential space between the periosteum and the galea aponeurotica of the scalp and is often associated with traumatic births, particularly those involving instrumental delivery, such as vacuum extraction or forceps use [1]. While subgaleal hemorrhage is well-documented for its potential to cause life-threatening complications due to significant blood loss, delayed subaponeurotic fluid collection (DSFC) is less common, typically benign, and self-limiting [2]. Unlike subgaleal hemorrhage, DSFC is not usually associated with acute symptoms at birth and tends to present later in infancy, often causing concern for caregivers due to their progressive nature. The differentiation between these conditions is crucial, as the management strategies and outcomes vary significantly.

This case highlights the diagnostic and management challenges of a DSFC in a 70-day-old infant following an instrumental vaginal delivery. The objective of this case report is to increase awareness of DSFC as a differential diagnosis in infants presenting with scalp swellings beyond the neonatal period. By documenting the clinical presentation, diagnostic considerations, and conservative management approach, this report aims to contribute to the limited literature on DSFC and emphasize the importance of distinguishing it from more serious conditions, such as subgaleal hemorrhage, which require prompt intervention. Additionally, this case underscores the role of careful birth history assessment and clinical vigilance in guiding appropriate management and avoiding unnecessary invasive procedures.

## Case presentation

A 70-day-old male infant was brought to the clinic by his parents with concerns regarding a progressively enlarging swelling on the vertex of the scalp. The swelling was first noticed on day 43 of life as a small, soft collection, which gradually increased in size over the following weeks. The infant remained otherwise asymptomatic, with no reported fever, irritability, feeding difficulties, or lethargy.

The infant was born at term following an uncomplicated pregnancy. The mode of delivery was vaginal delivery with the use of vacuum extraction. The labor was prolonged, and the APGAR scores at one and five minutes were within normal limits. No signs of scalp swelling were noted at birth. The newborn was discharged home without complications, and there was no history of trauma after birth.

Past medical history was unremarkable, with no prior hospitalizations, infections, or significant health concerns. There was no family history of bleeding disorders or congenital anomalies. Developmental milestones were appropriate for age, and the infant was up to date on routine immunizations.

**Figure 1:**
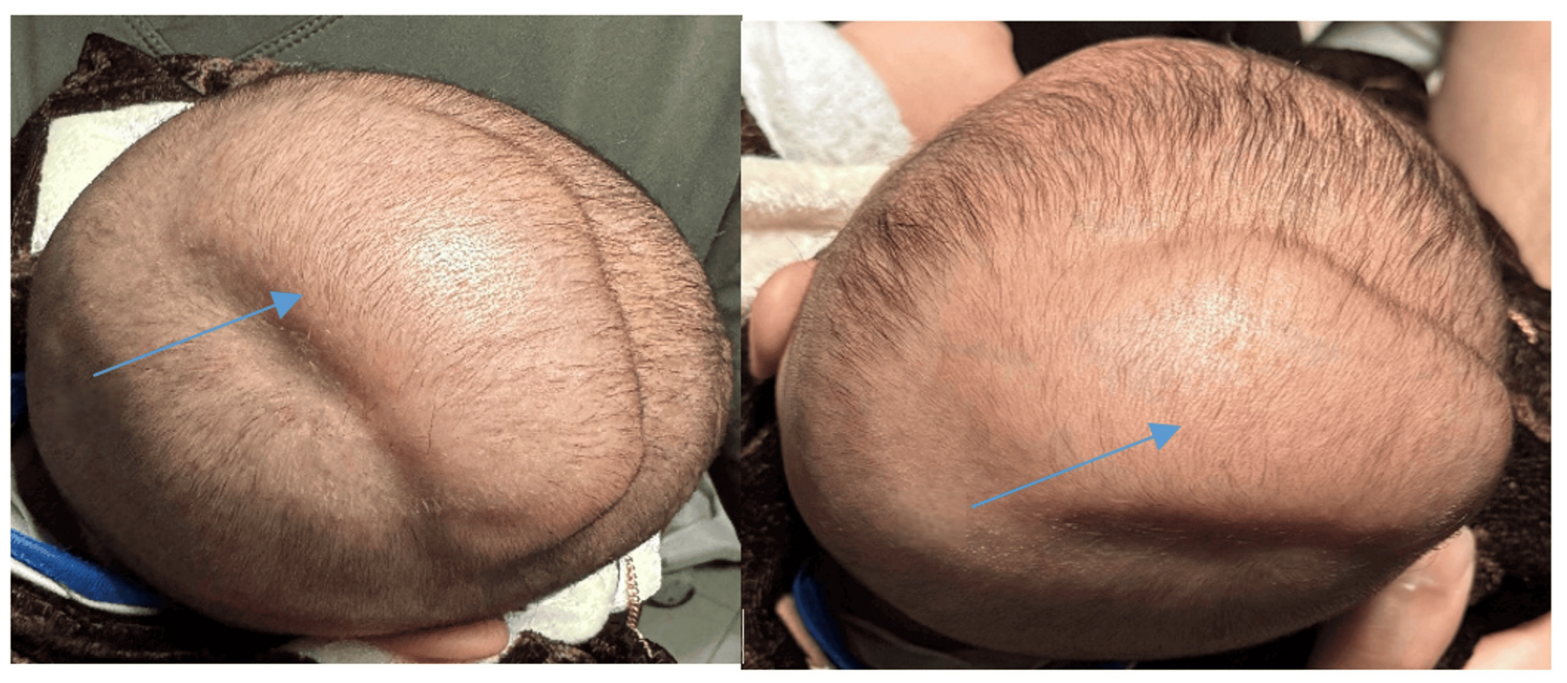
Clinical images showing a well-demarcated, soft, fluctuant swelling approximately over the vertex of the scalp.

On physical examination, the infant appeared well-nourished and alert, with normal vital signs. Local examination of the scalp revealed a soft, fluctuant, non-tender swelling overlying the vertex (Figure 1). The swelling was not associated with overlying skin changes such as erythema or warmth. It was non-pulsatile, and there were no signs of trauma, such as bruising or abrasions. The anterior fontanelle was soft and flat, with no evidence of increased intracranial pressure. The neurological examination was normal, with no focal deficits or abnormal reflexes.

There were no signs of systemic infection, and the infant was afebrile. Given the presentation and the absence of systemic symptoms, the initial differential diagnoses included (i) caput succedaneum, a benign, self-limiting condition that appears immediately after birth, crosses suture lines, and typically resolves within days, (ii) cephalohematoma, a subperiosteal hemorrhage confined to suture lines that does not increase in size after birth, (iii) subaponeurotic (subgaleal) hemorrhage, a more serious condition often linked to instrumental delivery, which can result in significant blood loss, and (iv) DSFC, a rare, self-limiting condition that occurs in older infants and usually resolves without complications.

**Figure 2:**
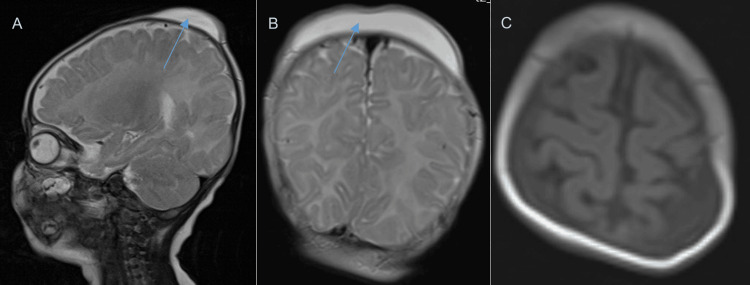
Sagittal (A) and Coronal (B) T2-weighted MRI scan revealing a well-defined hyperintense (fluid-filled) collection external to the skull in the subaponeurotic space.; Axial MRI scan (C) showing a localized hypointense scalp.

Further diagnostic assessment was considered to differentiate between these conditions. Basic laboratory investigations were done and showed normal results (Table 1). Ultrasound of the scalp is often useful in such cases, helping distinguish between fluid-filled collections, hemorrhages, or organized hematomas. However, in this case, an MRI scan of the brain was performed to evaluate the extent of the fluid collection and rule out any intracranial involvement. The sagittal and coronal T2-weighted MRI scan revealed a well-defined hyperintense (fluid-filled) collection external to the skull in the subaponeurotic space, confirming the absence of intracranial pathology (Figures 2A, 2B). The axial MRI scan further supported this diagnosis, showing a localized scalp swelling without evidence of midline shift, hydrocephalus, or significant parenchymal abnormalities (Figure 2C). These findings were consistent with DSFC, reinforcing the provisional diagnosis and justifying a conservative approach to management.

**Table 1:** Laboratory test results

Parameter	Result	Reference Range
Hemoglobin	15.1 g/dL	13.0-17.0 g/dL
Total Leukocyte Count	12 × 10^9/L	4.0-11.0 × 10^9/L
Platelets	130 × 10^9/L	150-450 × 10^9/L
Serum Sodium	132 mmol/L	135-145 mmol/L
Serum Potassium	4.68 mmol/L	3.5-5.0 mmol/L
Serum Chloride	98.7 mmol/L	98-107 mmol/L
Urea	44 mg/dL	15-45 mg/dL
Creatinine	0.98 mg/dL	0.6-1.3 mg/dL
Activated Partial Thromboplastin Time (aPTT)	30.0 seconds	25-35 seconds
Prothrombin Time (PT)	11.0 seconds	10-13 seconds
International Normalized Ratio (INR)	1.0	0.8-1.2
C-Reactive Protein	<5 mg/L	<5 mg/L

Based on the clinical presentation, absence of systemic symptoms, and progressive nature of the swelling, a provisional diagnosis of DSFC was made. This rare entity is typically self-resolving and requires no invasive intervention unless complications arise. The infant was advised conservative management, including careful monitoring for any signs of infection, rapid swelling progression, or neurological deterioration. The parents were educated on warning signs such as fever, irritability, vomiting, or lethargy, which would necessitate immediate medical attention.

A follow-up appointment was scheduled in four weeks to monitor the resolution of the swelling. The use of MRI in this case provided valuable confirmation of the fluid collection's location and nature, reinforcing the decision to manage the infant conservatively while ensuring close monitoring for any potential complications.

Follow-up and outcome

At the two-month follow-up, the infant remained clinically well with no neurological symptoms or developmental concerns. On physical examination, the previously noted scalp swelling had completely resolved without residual fluctuation or deformity. There were no signs of infection, recurrence, or complications.

**Figure 3:**
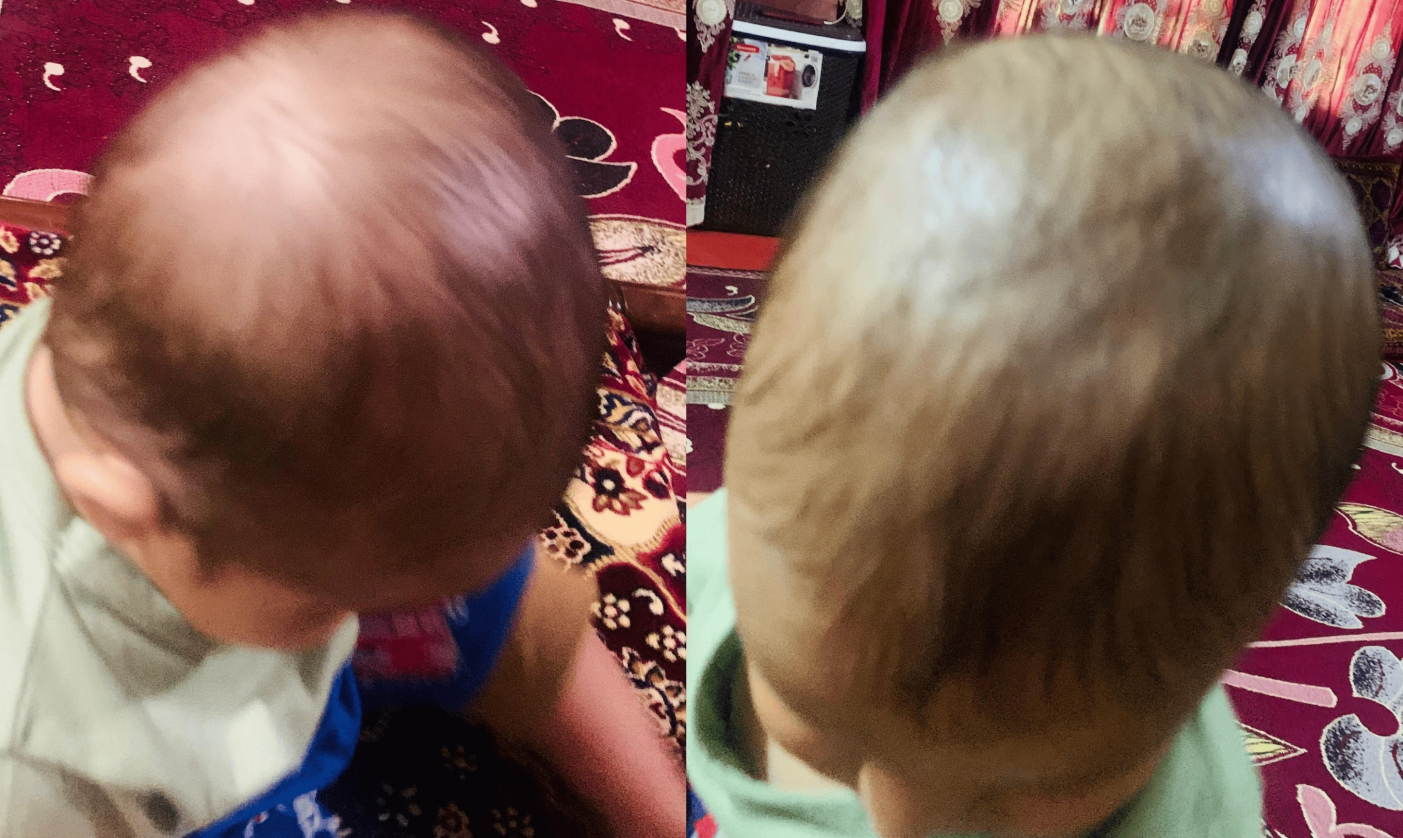
Follow-up clinical images showing complete resolution of the previously observed subaponeurotic scalp swelling, with normal scalp contour and no residual collection.

Clinical photographs obtained at follow-up demonstrate complete resolution of the DSFC (Figure 3). This outcome further supports the benign, self-limiting nature of DSFC and validates the conservative management approach adopted in this case.

## Discussion

This case is significant due to the rarity and atypical presentation of DSFC in an infant beyond the neonatal period. While subaponeurotic collections are commonly associated with immediate postnatal trauma [3], this case demonstrates a delayed onset nearly six weeks after birth, in the absence of overt trauma or systemic symptoms. The progressive enlargement of the scalp swelling without signs of infection or neurological compromise posed a diagnostic challenge, highlighting the importance of considering DSFC in the differential diagnosis of scalp swellings in infants. Additionally, the conservative management approach adopted in this case reinforces the self-limiting nature of DSFC, contrasting with more aggressive treatments required for similar conditions like subgaleal hemorrhage. 

When compared to existing literature, this case aligns with previous reports describing DSFC as a benign, self-resolving condition primarily occurring in infants with a history of instrumental delivery, such as vacuum extraction or forceps use [4]. Similar cases have reported the absence of systemic symptoms and the presence of a soft, fluctuant swelling that increases gradually over time [3]. However, this case differs slightly in the delayed onset of symptoms and the progressive nature of the swelling, which persisted beyond the typical resolution period described in the literature.

The pathophysiology of DSFC involves the accumulation of serous fluid in the potential space between the galea aponeurotica and the periosteum of the skull [5]. This space, which normally contains minimal fluid, can become distended due to minor birth trauma, particularly in cases of instrumental deliveries where negative pressure or shear forces disrupt small blood vessels. Unlike subgaleal hemorrhage, which involves active bleeding and can lead to significant blood loss and hemodynamic instability, DSFC typically consists of clear, non-bloody fluid and lacks the acute, life-threatening features of hemorrhagic collections. The delayed presentation in DSFC may result from slow fluid accumulation or delayed recognition, especially in asymptomatic infants.

The diagnostic process in this case involved careful consideration of various differential diagnoses, including caput succedaneum, cephalohematoma, and subgaleal hemorrhage. Caput succedaneum, which crosses suture lines, typically resolves within a few days after birth and is unlikely to present as a delayed, progressive swelling [1-3]. Cephalohematomas are confined by suture lines and do not increase in size postnatally. Subgaleal hemorrhage, while sharing anatomical similarities with DSFC, usually presents acutely with signs of hypovolemia or shock. The absence of systemic signs, normal neurological findings, and the soft, fluctuant nature of the swelling led to the consideration of DSFC.

While ultrasound is often the first-line imaging modality in such cases due to its ability to distinguish between fluid-filled collections, hematomas, and soft tissue edema, an MRI scan was performed in this case to further evaluate the extent of the fluid collection and rule out any intracranial pathology. The T2-weighted MRI images confirmed a well-demarcated hyperintense collection external to the skull in the subaponeurotic space, with no underlying parenchymal abnormalities, midline shift, or hydrocephalus. MRI is particularly valuable in atypical or progressive cases as it provides detailed visualization of the fluid collection's composition, location, and relationship to surrounding structures, helping to differentiate DSFC from subgaleal hemorrhage [5]. The imaging findings in this case reinforced the diagnosis of DSFC and justified a conservative management approach. Potential pitfalls in diagnosis include mistaking DSFC for a more serious condition like a subgaleal hemorrhage, underscoring the importance of thorough birth history, clinical examination, and appropriate imaging to ensure accurate diagnosis and optimal management.

The management approach in this case was conservative, based on the absence of systemic symptoms, neurological deficits, or signs of infection. Conservative management is consistent with the literature on DSFC, which suggests that it is a self-limiting condition, with spontaneous resolution of fluid collections within four to eight weeks without the need for invasive interventions such as aspiration or surgical drainage, and complications are rare unless secondary infection or rapid expansion occurs [1-3,5,6]. In rare instances, if secondary infection or persistent swelling occurs, further investigation and intervention may be necessary.

In this case, the expectation of complete resolution aligns with existing knowledge, reinforcing the benign nature of the condition. The lack of long-term complications or recurrence in similar cases supports the decision for conservative management and highlights the favorable prognosis associated with DSFC [6].

This approach minimizes the risk of introducing infection and avoids unnecessary trauma. In contrast, conditions like subgaleal hemorrhage often require urgent medical intervention due to the risk of significant blood loss and shock. The decision to monitor the infant closely with scheduled follow-ups reflects the standard of care for DSFC, emphasizing the importance of parental education regarding warning signs like fever, irritability, or rapid swelling progression that could indicate complications [7].

This case provides several important lessons for clinical practice. It underscores the need for early recognition of DSFC as a differential diagnosis in infants presenting with scalp swellings beyond the neonatal period. It also highlights the importance of avoiding unnecessary interventions, as conservative management is typically sufficient in the absence of complications. Furthermore, the case emphasizes the role of parental education in monitoring for potential complications and ensuring appropriate follow-up. By documenting this case, we contribute to the limited literature on DSFC and reinforce the importance of distinguishing it from more serious conditions like subgaleal hemorrhage, ultimately guiding clinicians toward more accurate diagnoses and appropriate management strategies.

## Conclusions

This case highlights the importance of recognizing DSFC as a rare but benign cause of progressive scalp swelling in infants, particularly following instrumental vaginal delivery. The absence of systemic symptoms, normal neurological examination, and characteristic fluctuant swelling supported the diagnosis, allowing for conservative management without the need for invasive interventions. This case underscores the need for thorough birth history assessment and careful clinical evaluation to differentiate DSFC from more serious conditions like subgaleal hemorrhage or cephalohematoma. Educating parents about warning signs and ensuring appropriate follow-up are critical components of management, as most cases resolve spontaneously without complications. By documenting this case, we aim to enhance awareness of DSFC and its favorable prognosis, contributing to more effective and cautious clinical decision-making in similar presentations.
